# Nanobiocide Based-Silver Nanomaterials Upon Coronaviruses: Approaches for Preventing Viral Infections

**DOI:** 10.1186/s11671-021-03558-3

**Published:** 2021-06-06

**Authors:** Kamyar Khoshnevisan, Hassan Maleki, Hadi Baharifar

**Affiliations:** 1 Biosensor Research Center, Endocrinology and Metabolism Molecular-Cellular Sciences Institute, Tehran University of Medical Sciences, Tehran, 1411713137 Iran; 2 Endocrinology and Metabolism Research Center, Endocrinology and Metabolism Clinical Sciences Institute, Tehran University of Medical Sciences, Tehran, 1411713137 Iran; 3 Nano Drug Delivery Research Center, Health Technology Institute, Kermanshah University of Medical Sciences, Kermanshah, Iran; 4 Department of Medical Nanotechnology, Applied Biophotonics Research Center, Science and Research Branch, Islamic Azad University, Tehran, 1477893855 Iran

**Keywords:** Nanobiocides, Coronaviruses, Antiviral activity, Silver nanomaterials, Inhibitory mechanisms, Viral infection, COVID-19

## Abstract

**Abstract:**

The effectiveness of silver nanomaterials (AgNMs), as antiviral agents, has been confirmed in humans against many different types of viruses. Nanobiocides-based AgNMs can be effectively applied to eliminate coronaviruses (CoVs), as the cause of various diseases in animals and humans, particularly the fatal human respiratory infections. Mostly, these NMs act effectively against CoVs, thanks to the NMs’ fundamental anti-viral structures like reactive oxygen species (ROS), and photo-dynamic and photo-thermal abilities. Particularly, the antiviral activity of AgNMs is clarified under three inhibitory mechanisms including viral entry limitation, attachment inhibition, and viral replication limitation. It is believed that nanobiocide with other possible materials such as TiO_2_, silica and, carbon NMs exclusively nano-graphene materials can emerge as a more effective disinfectant for long-term stability with low toxicity than common disinfectants. Nanobiocides also can be applied for the prevention and treatment of viral infections specifically against COVID-19.

**Graphic Abstract:**

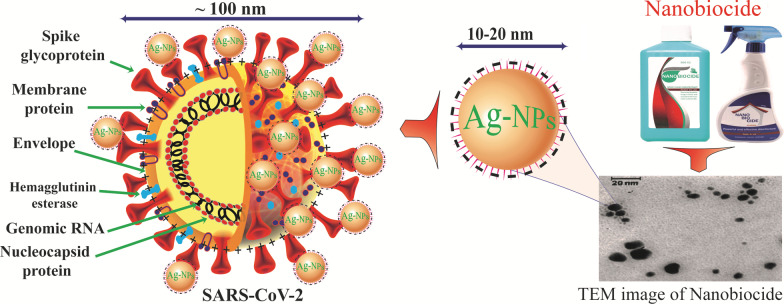

## Introduction

Virus infections are a major global health challenge, particularly because the emergence of resistant and unknown viral strains and the complications related to extended use reduce the usefulness of postoperative antiviral therapies against pathogenic viruses [1–3]. Based on the world health organization (WHO) reports, humans encountered several viral infections from the beginning of the twenty-first century. Severe acute respiratory syndrome coronavirus (SARS-CoV), H1N1 influenza, and Middle East respiratory syndrome coronavirus (MERS-CoV) started in 2002, 2009, and 2012 respectively in different countries (Centers for Disease Control and Prevention [CDC], WHO). Lately, another viral pandemic has occurred that is called "COVID-19," or "coronavirus disease 2019". Being very contagious, the new virus has influenced human lives, the global economy, and people's livelihoods [4, 5].

Many scientists and pharmaceutical companies have commenced discovering a well-organized and effective way to protect human lives from the two arising epidemic infectious diseases caused by coronaviruses, such as SARS and MERS in the last two decades. Small molecules, common antiviral drugs, antibody-based drugs are the most promising tools for the treatment of viral infections [6]. In the case of new viral infections, efficient medication development desires plenty of studies, cost, and time. Therefore, in the absence of efficient treatment protocols and therapeutics, prevention of the viruses is one of the best ways to reduce virus infections [7]. Prevention of the viruses could be reached through different approaches. In the case of respiratory-associated viral infections, keeping a safe distance, and removing or/and neutralizing virus particles from the surface reduces contamination risk [8]. Most of the viruses could be removed or neutralized using different disinfectants such as alcohol and heat. Although traditional disinfectants are capable of removing and neutralizing many viruses, they cannot be used by everyone in all conditions. Therefore, a great motivation has been shaped by scientists for designing and developing new disinfectants as a giant step in the prevention of infectious diseases spread [9].

**Fig. 1:**
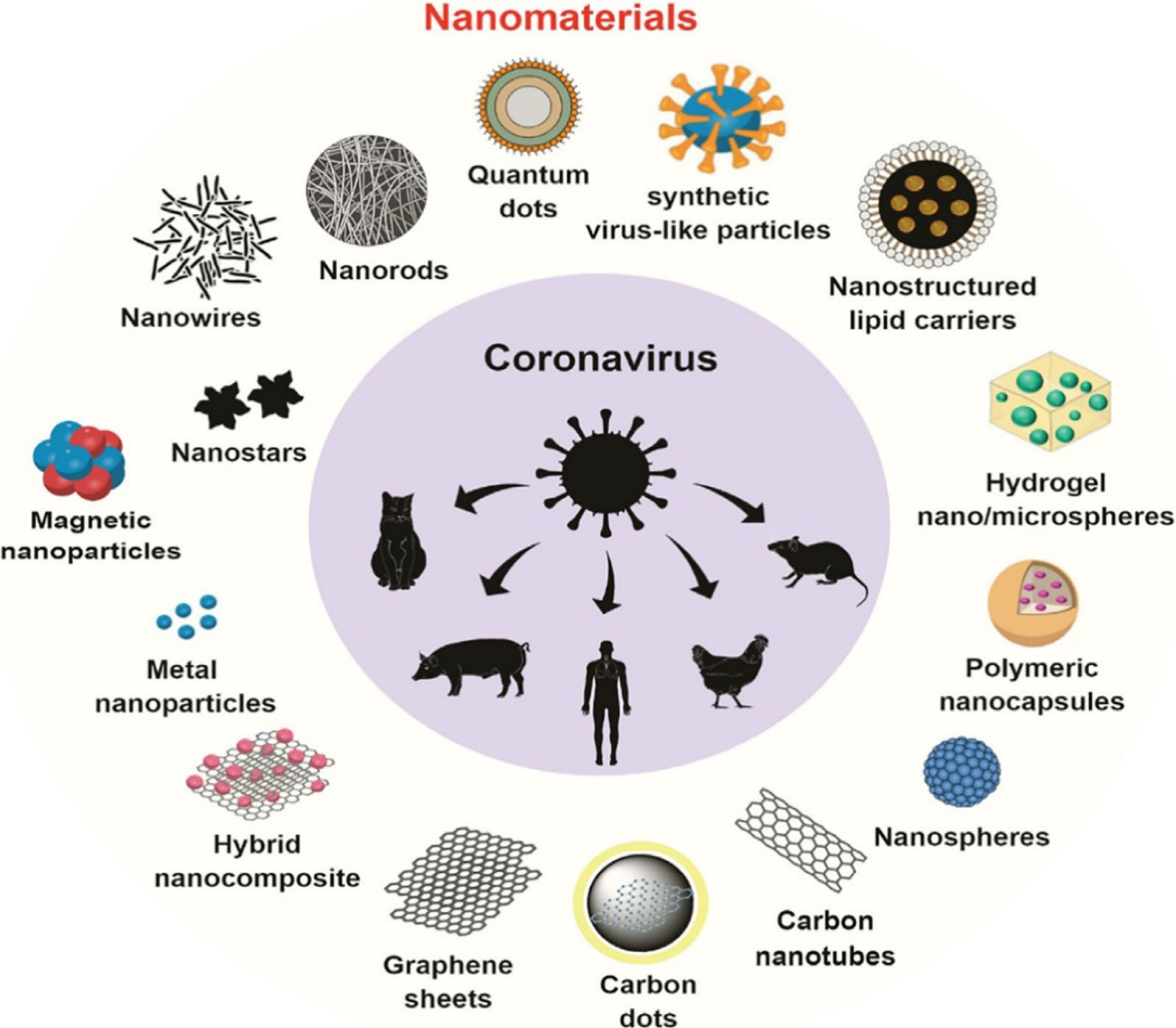
The most common nanomaterials with antiviral activity against animal/human COVs including the novel SARS‐CoV‐2. Reproduced from Ref. [24]

Regarding the capability of all new science and technologies, nanotechnology offers powerful versatile tools for the investigation, detection, prevention, and treatment of viral infections [10]. The dimension of viruses is commonly in the range of nano-scale and hence, the nanomedicine field is heading to investigate the nanoparticle uptake in cells, and thus the approaches and values from the synthesis of nanoparticles with cells present the mechanisms that are before viral infections [11]. Gold nanoparticles immunochromatographic [12] and electrochemical tests have been under development as a rapid and cost-effective screening method for viral infection detection [13]. The application of silver nanomaterials (AgNMs) has been regarded as a new approach against resistant viral and bacterial strains [14–16]. AgNMs can provide an important role in controlling and treatment of unknown infectious diseases. Ag nanoparticles (NPs), as one of the most familiar NPs, have been extensively applied to detect, neutralize and treat viral infections. AgNMs can be synthesized simply by various methods such as green (biological), chemical, and physical [17]. It should be mentioned that AgNMs on a large scale should be applied with caution. Some studies have described the potential toxic effect of silver-based materials and many barriers to the ecosystem [18, 19]. The particles result in the generation of reactive oxygen species (ROS) in vital cells at specific concentrations and sizes [20]. Using particles for medical purposes must be done by considering both benefits and limitations. Fortunately, AgNMs have undergone many research and development procedures to find out a novel strategy to either defeat or ameliorate the severity of the infection [1, 21, 22]. Up to now, the effectiveness of AgNMs as an antiviral agent has been verified in humans against many viruses, including human immunodeficiency virus, hepatitis B virus, herpes simplex virus, respiratory syncytial virus, poliovirus, adenovirus, and monkeypox virus [20]. Recently, Das et al. independently of our group reviewed the possibility of AgNMs as therapeutic agents against coronavirus and ultimately they suggested applying nanomaterials coating in personal protection equipment for the prevention of infection-associated coronaviruses [23]. In the same case, Carvalho et al. reported the most common NMs with anti‐coronavirus activity against animal and human COVs (Fig. 1) in a systematic review study [24]. It is worth bearing in mind, nanobiocides-based materials are metal and metal oxides, engineered/synthesized NMs, and natural antibacterial substances showing strong antimicrobial activity through diverse mechanisms [25].

As alluded to above, many types of NMs have been applied against COVs, however, the comprehensive investigation and inspection of engineered NMs like nanobiocides still remained. Therefore, we aimed to focus on summary points of nanobiocides-based AgNMs effects on COVID-19 as a novel prevention approach.

## Coronaviruses/Family

In recent years, the CoV outbreak has spread all over the world causing epidemic and pandemic outbreaks of acute respiratory and infectious diseases (http://www.cdc.gov/coronavirus/). Coronavirus has four genera containing alpha, beta, gamma, and delta. Seven types of *betacoronaviruses* family are potent to infect human and three of them cause severe symptoms (i.e. ARS-CoV, MERS-CoV, and SARS-CoV-2). Bats and rodents are the natural reservoirs of different *betacoronaviruses* subgenera [26].

The estimated size of CoVs is around 100 nm. CoVs, enveloped single-strand positive-sense RNA viruses, are recognized by club-like spikes that stick out from their surface, an abnormally large RNA genome, and have a distinctive replication plan [27]. The virus genomes encoded different structural and non-structural proteins. The structural proteins are spike (s), membrane (m), envelope (e), and nucleocapsid (n). CoV bonded to angiotensin-converting enzyme 2 (ACE2) receptor of the host cells using its S protein. S protein has two subunits and as a virulence factor has been applied for antibody base treatment and vaccine development [28]. Despite numerous research, unfortunately, there is no efficient treatment or vaccination for CoVs infection yet. Therefore the majority of CoVs, such as SARS-CoV and MERS-CoV, can cause fatal infection in humans [29].

## Mechanism of Action

The AgNPs mechanism for viral limitation varies from virus to virus. For instance, the commercial fabricated AgNPs with antiviral efficacy have been applied against HIV-1, HSV-2, avian influenza A virus, Peste des petits ruminants' (PPR) virus, and some resistant strains, such as IIIB, Eli, Beni, 96USSN20, and SaquinavirRV [21]. Typically, envelope glycoprotein (gp120) interaction with host receptor CD4 and related coreceptor triggers major conformational changes in the virus and causes the releases of viral core into the cytoplasm. The results revealed that AgNPs deter CD4-dependent virion binding, amalgamation, and pathogenesis by linking with viral gp120 in both cell-free and cell-associated viruses [30]. In the case of HSV-2, AgNPs interact with the sulfhydryl group, existing on the membrane glycoproteins, to overcome the limitations of antiviral drugs and avoid viral internalization. Concerning the PPR virus, AgNPs prevent spoiling viral replication and entry by interacting with virion surface and core protein. [22, 31]. In the case of CoVs, graphene oxide (GO) sheets and with AgNPs (GO-Ag) were synthesized to be used against enveloped and non-enveloped feline coronavirus (FCoV) with and without an envelope and infectious bursal disease virus (IBDV). The results obtained from this study revealed that Go-Ag reduced, respectively, the FCoV and IBDV infections by 25% and 23%; whereas, GO only reduced FCoV infections by 13% while showing no antiviral activity against the IBDV [32]. Thus, the antiviral activities of AgNMs are explained under the following circumstances:

## Viral entry limitation, attachment inhibition, and viral replication limitation

**Fig. 2:**
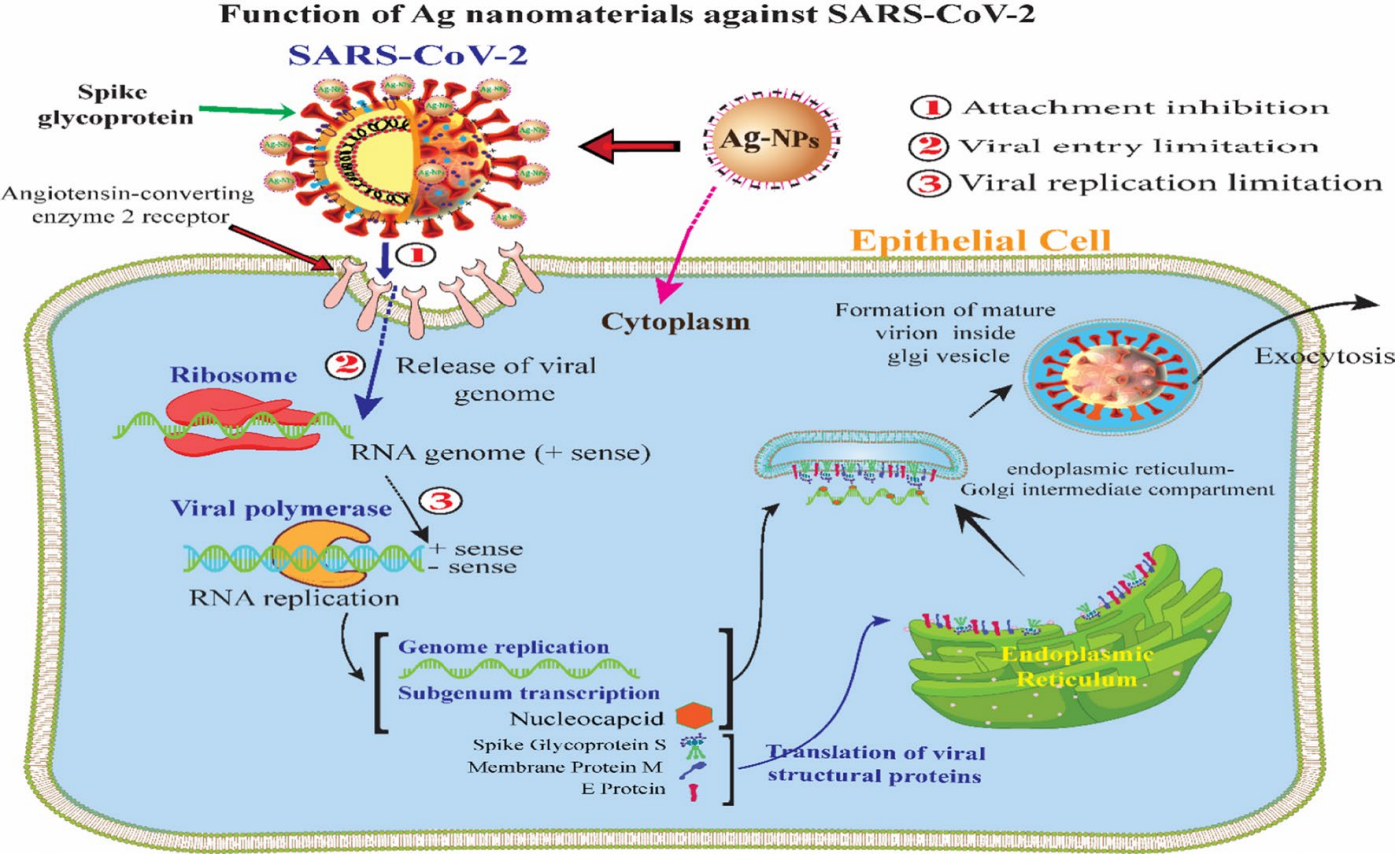
The conceptual design of the function of Ag NMs for antiviral activity

Such antiviral nanomaterials can specifically inhibit viral infectivity irreversibly by blocking the interaction between SARS-CoV-2 spike protein with the angiotensin-converting enzyme-2 (ACE2) receptor [33]. AgNPs can link to viral surface proteins rich in sulfhydryl groups and cleave the disulfide bonds to disrupt the protein which leads to impaired viral binding to the target cell receptor [34, 35]. It can be assumed that the main antiviral mechanism of action of AgNPs against SARS-CoV-2 is effectively inhibited viral entry step by either preventing viral attachment or interfere with viral entry, or by damaging the surface proteins to disrupt the structural integrity of virions (Fig. 2). Besides, AgNPs can enter into cell cytoplasm and intracellular antiviral action by interacting with observed viral nucleic acids, and possibly due to the limitation of viral replication leading to inhibit serial viral infection of newly produced virus from infected cells to uninfected cells [36]. Further studies should be conducted to more precisely explore the antiviral action of AgNPs on SARS-CoV-2 and clarify it in depth consequently.

## Impact of Nanobiocides on COVs

**Fig. 3:**
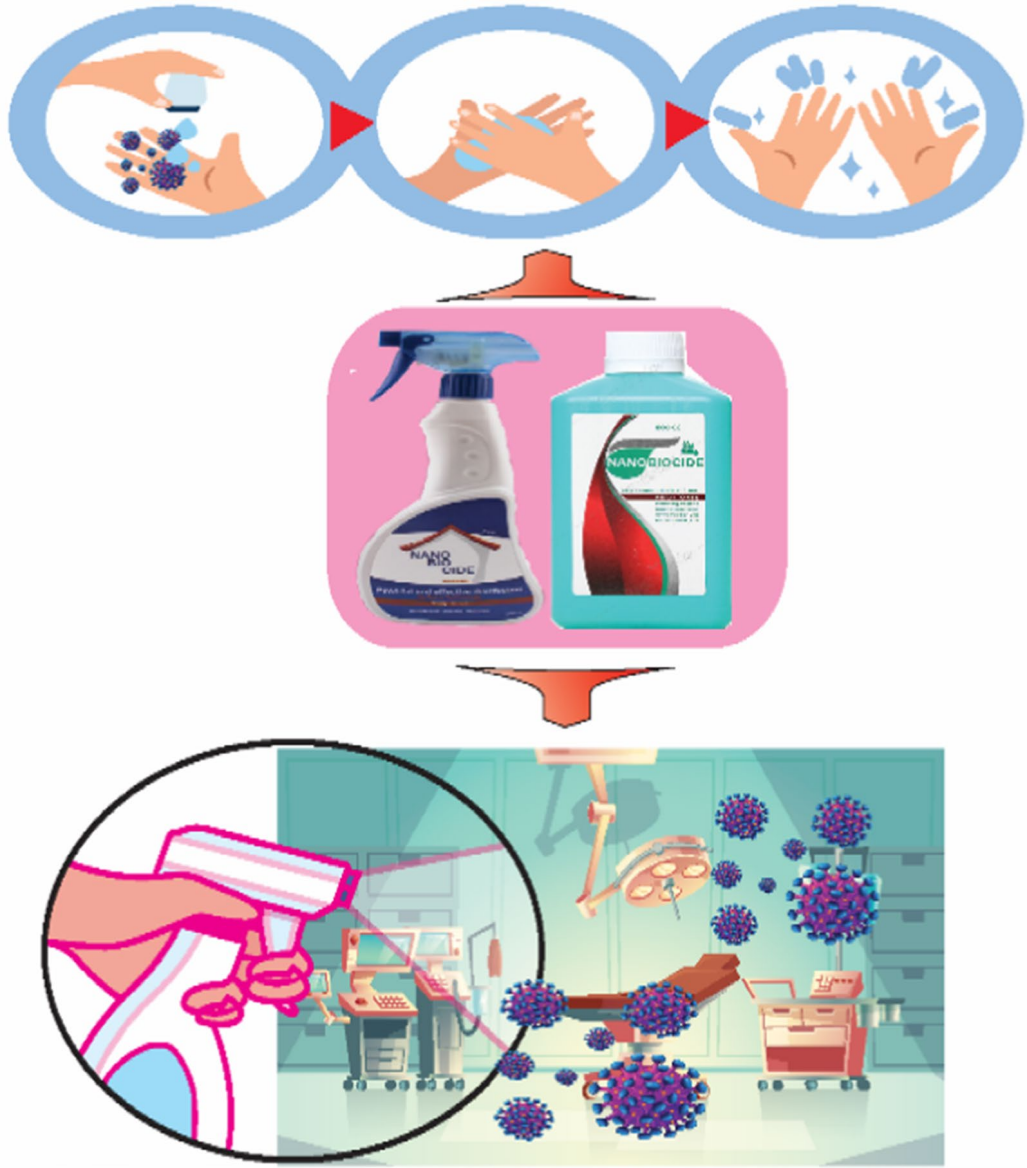
The application of nanobiocides as the sanitizer and disinfectant for hand washing and healthcare systems

The use of reliable, low-cost, and rapid virucidal agents with long-term efficiency for SARS-CoV-2 prevention is the foremost priority. Nanobiocides, including metal nanoparticles and engineered nanomaterials, are effectively stabilized by using ionic surfactants typically with a negative charge to present high antiviral activity and stability in water. The surfactant molecule is dispersed in a colloidal liquid to enhance the stability for globular micelle reaching in nano-size [25]. Colloidal stability and continued circulation time are the strengths of the surfactant molecule [37]. Nanobiocides (e.g. AgNPs along with ionic surfactant) are commercial products of stable colloid, including 150 ppm Ag-NPs, used for surface disinfection from viruses, e.g. in hand sanitizers (Fig. 3) [38, 39]. In one study, cytotoxicity tests for nanobiocide were carried out in white albino rabbits. No sign of gross toxicity, pharmacological side effects, and abnormal behavior were observed in the animal-based Draize dermal irritation scoring system (DDIS), inhalation toxicity and irritation testing, etc. [38]. Additionally, the antiviral effect of AgNPs against SARS-CoV-2 has been confirmed in a size- and concentration-dependent manner [36]. The Effective NPs were found to be about 20 nm at concentrations ranging between 50 and 100 ppm, however, the high dosage (< 200 ppm) may cause a cytotoxic effect [36].

A recent study by Jeremiah et al. found that [36], naked AgNPs with diameter 2–15 nm were effective in inhibiting extracellular SARS-CoV-2 at concentrations ranging between 1 and 10 ppm while cytotoxic effect was observed at concentrations of 20 ppm and above on mammalian cells.

The antiviral effects of AgNPs are size and concentration-dependent which depend on the cell type and also the type of AgNPs. Smaller particles have a higher toxic potential due to the greater surface area of interaction with the bound protein. Most studies have observed the antiviral efficacy of AgNPs at concentrations ranging between 10 and 100 ppm [1]. It is believed that nanobiocides containing AgNPs could be potentially applied as antiviral agents for the prevention and treatment of viral infections without any obvious adverse effects on humans and animals. Generally, These NMs can effectively act as a disinfectant against CoVs, owing to the NMs’ fundamental anti-viral features like reactive oxygen species (ROS) generation and photo-dynamic and photo-thermal abilities. Likewise, NMS-loaded lipid-based biodegradables, such as ionic polymeric surfactants, can be applied to eliminate the side effects of metallic NMs on humans and the environment [10]. Nanobiocides could interact with CoVs via ionic bonds on spike glycoprotein or protein-membrane surface.

Other metal nanoparticles composed of gold (Au) titanium (Ti), and zinc (Zn) have already shown great results against different types of viruses mainly thanks to their various antimicrobial activities that could be used on inanimate and nonbiological surfaces to efficiently control the ongoing COVID-19 pandemic [40].

In recent years, Silica nanoparticles have been exclusively applied to control viral transduction [41], deliver antiviral drugs [42], and so on. Besides, these NPs can be also used for binding to oligonucleotides to providing a novel platform for vaccine delivery [43].

Applying functionalized silica nanoparticles revealed the attachment of cell receptors to the virus envelope which was inhibited by a strong virus-nanoparticle interaction owning to the similarity in their hydrophobic/hydrophilic characters [41]. Moreover, mesoporous silica nanoparticles were developed as a suitable, safe, and effective candidate for encapsulation, protection, and delivery drugs and nucleic acids [43]. Such nanoparticle-based vaccines offer a promising approach for an effective delivery system for DNA/RNA vaccines along with high payloads, tunable sizes, tailorable surface properties, controllable release kinetics, and improved stability [44]. 

Likewise, organic nanoparticles for delivering antivirals as nanoencapsulated drugs have been applied to improve drug bioavailability and targeted antiviral activity and also to promote efficient drug delivery that could be alternatives to the development of safer treatments for COVID-19 and other viral diseases. Nanoencapsulated drugs through activating/deactivating intracellular mechanisms may be more efficient to make irreversible destruction to viruses and interfere with the virus' reproduction cycle [45].

Graphene also showed a good capacity for viral inhibition. Graphene-based material has been revealed that can hinder the entry and replication of enveloped RNA virus (coronavirus) and DNA virus (herpesvirus) in their target cells [46, 47]. Studies indicated negatively charged GO association with positively charged viral lipid tails leads to aggregation and rupture of lipid membranes [32].

**Fig. 4:**
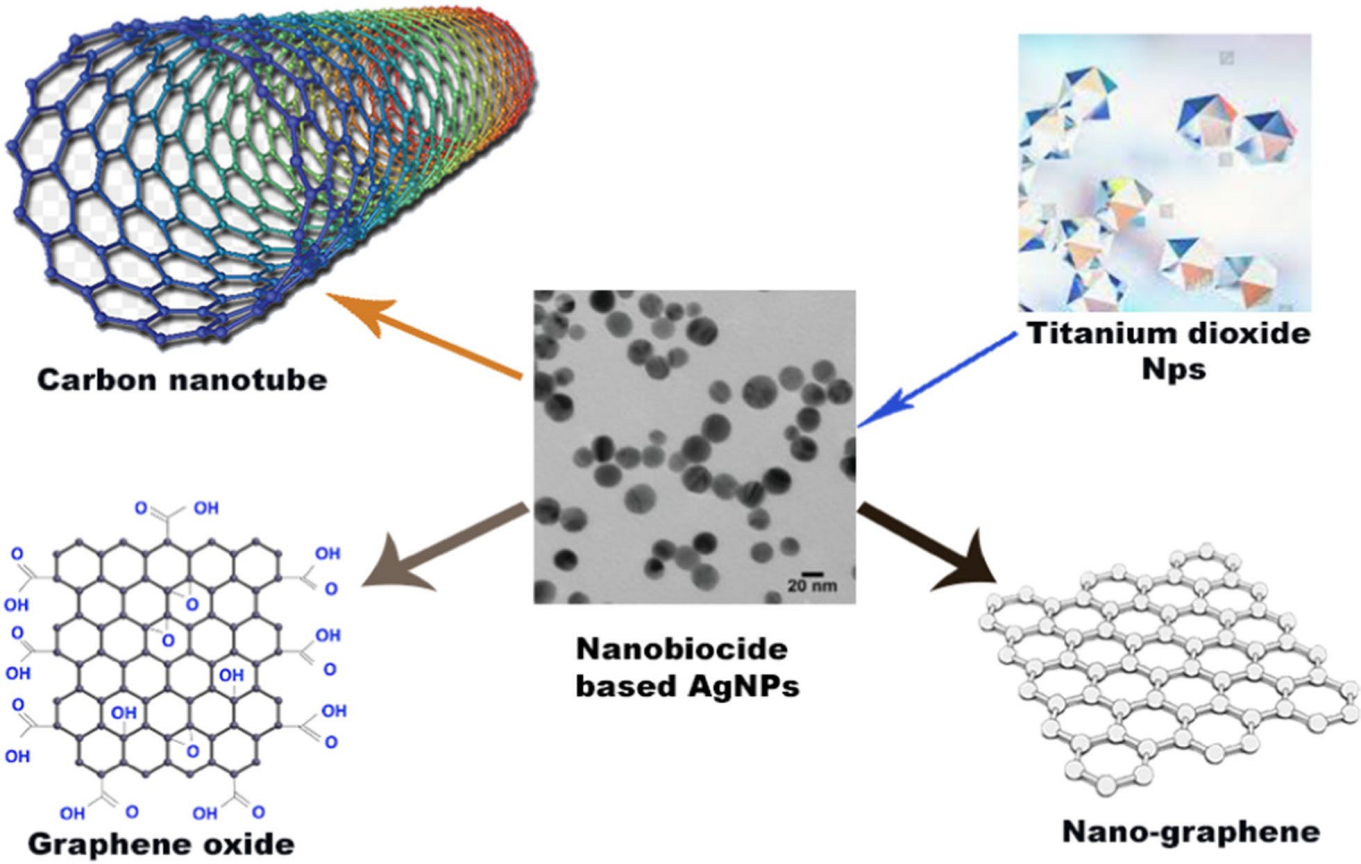
Nanobiocides based AgNPs merged with carbon NMs and TiO_2_

Overall, nanobiocides could help the fight against COVID-19 especially through avoiding viral contamination and propagation by providing infection-safe individual protective equipment to augment the safety of medical workers and development of efficient antiviral disinfectants, sanitizers, and surface coatings for frequently touched surfaces, which can inactivate the virus and prevent its spread. Nanobiocides based AgNMs along with other nanomaterials such as TiO_2_ and carbon nanomaterials exclusively nano-graphene materials can produce a more efficient disinfectant for long-term stability and low toxicity compared to common disinfectant (Fig. 4).

## Perspectives and Conclusions

**Fig. 5:**
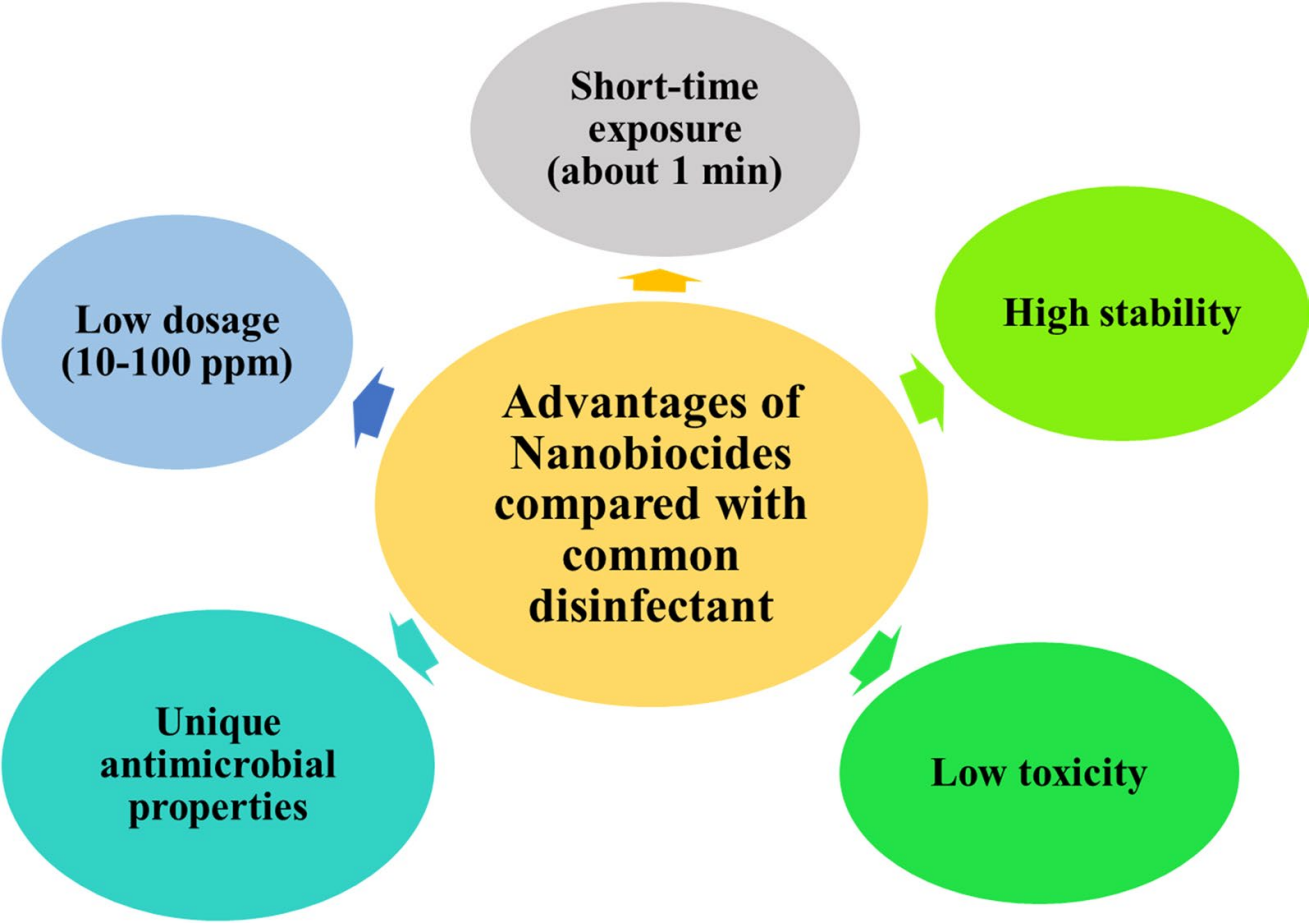
An overview of nanobiocides’ advantages

Thanks to the nanobiocides features, including the limitation of viral entry and replication, they have great antiviral efficiency and provide a simple way to eliminate many different types of viruses. Likewise, nanobiocides based-AgNMs can be extensively applied as a disinfectant for sensitive and non-sensitive surfaces. Also, low dosage (10–100 ppm), short-time exposure (about 1 min), high stability, and low toxicity make them superior over the common disinfectants, such as ethanol, sodium hypochlorite, and hydrogen peroxide. An overview of nanobiocides’ advantages was displayed in Fig. 5. It also seems that nanobiocides can be employed for fighting acute viral infections [39]. Currently, due to concern over the COVID-19 pandemic all around the world, the nanobiocides can efficiently be applied to deactivate the viral activity of CoVs. However, it also seems that the nanobiocides along with other nanomaterials can be applied as a more effective and high-quality disinfectant for long-term stability and low toxicity. In conclusion, the nanobiocides have opened a door for performing studies on novel antiviral agents and many other goals.

We hope that our investigation could have successfully addressed the role of nanobiocides as a unique antimicrobial agent which can aid scientists and researchers with their valuable insights to conquer the obstacles related to the SARS‐CoV‐2 virus control. We also hope that these engineered NMs including metal and metal oxide NPs give us a suitable direction to provide exclusive antiviral therapies and avoid forthcoming pandemics close to the current COVID‐19.

## Data Availability

No data and materials are included in this article.
